# Role of Arsenic During Aluminum Droplet Etching of Nanoholes in AlGaAs

**DOI:** 10.1186/s11671-016-1648-6

**Published:** 2016-09-26

**Authors:** Christian Heyn, Michel Zocher, Sandra Schnüll, Wolfgang Hansen

**Affiliations:** Institut für Nanostruktur- und Festkörperphysik, Center for Hybrid Nanostructures (CHYN), Universität Hamburg, Jungiusstraße 11, Hamburg, D-20355 Germany

**Keywords:** Semiconductor, Nanostructuring, Self-assembly, Droplet etching

## Abstract

Self-assembled nanoholes are drilled into (001) AlGaAs surfaces during molecular beam epitaxy (MBE) using local droplet etching (LDE) with Al droplets. It is known that this process requires a small amount of background arsenic for droplet material removal. The present work demonstrates that the As background can be supplied by both a small As flux to the surface as well as by the topmost As layer in an As-terminated surface reconstruction acting as a reservoir. We study the temperature-dependent evaporation of the As topmost layer with in situ electron diffraction and determine an activation energy of 2.49 eV. After thermal removal of the As topmost layer droplet etching is studied under well-defined As supply. We observe with decreasing As flux four regimes: planar growth, uniform nanoholes, non-uniform holes, and droplet conservation. The influence of the As supply is discussed quantitatively on the basis of a kinetic rate model.

## Background

The integration of metal droplet-based processes into semiconductor molecular beam epitaxy (MBE) represents a qualitative extension of the MBE method and allows the self-assembled creation of various III/V semiconductor nanostructures [1–14]. Dependent on substrate temperature and group V element background pressure, either material is deposited or removed from the substrate surface. Processes adding material to the substrate are called droplet epitaxy [1–7] and those with material removal nanodrilling or local droplet etching (LDE) [8–14].

**Fig. 1:**
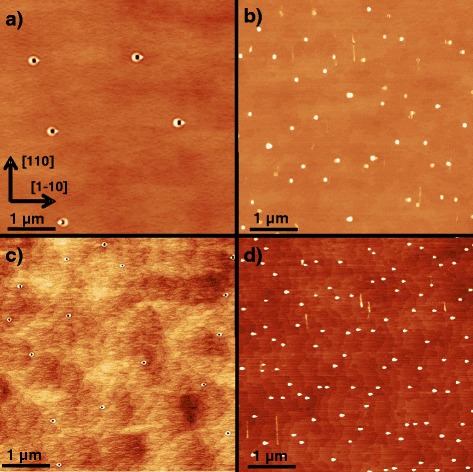
AFM images of AlGaAs surfaces after LDE with Al droplets at varied parameters. **a** Sample with *T*=605 °C, *θ*_Al_=1.4 ML, and *P*_As_≃6×10^−8^ Torr. **b** Like **a** but with an additional 670 °C pre-growth overheating step. **c** Sample with *T*=550 °C, fully minimized *P*_As_≃1×10^−10^ Torr, *θ*_Al_=1.0 ML, and no overheating. **d** Like **c** but with overheating

This study focuses on LDE with Al droplets to form low-density nanoholes in (001) AlGaAs surfaces. An example is shown in Fig. 1a. The nanohole depth can be tuned by the amount of deposited droplet material and the process temperature from 1 nm up to more than 100 nm [15]. By hole filling with a material different from the substrate, droplet-etched nanoholes represent an interesting template for the self-assembled creation of, e.g., strain-free GaAs quantum dots [13] or nanopillars for thermoelectrics [14].

LDE is based on the the transformation of deposited nanodroplets into nanoholes. The central process for hole formation is diffusion of arsenic from the crystalline AlGaAs substrate into an Al droplet driven by the concentration gradient [16]. As a consequence, the substrate liquefies at the interface to a droplet. A second important process is the removal of the liquid material from the initial droplet position. Previous studies indicated already that a small amount of background arsenic is essential for this [17, 18]. Without background As, the initial droplets are conserved and no holes are formed.

The present paper demonstrates that the As background required for droplet etching can be supplied not only by an As flux to the surface but also by an As-rich surface reconstruction acting as a reservoir. After controlled evaporation of the As reservoir, the influence of the As flux on local droplet etching is studied under well-defined conditions.

## Methods

The samples are fabricated in a solid-source molecular beam epitaxy chamber equipped with a valved-cracker source for As _4_ evaporation. The initial surface for droplet etching is Al _*x*_*Ga*_1−*x*_As (*x* =0.35) grown on (001) GaAs wafers at *T*=600 °C and an As _4_ flux corresponding to a flux gauge reading of about *P*_As_=1×10^−5^ Torr.

Local droplet etching is performed in three steps: pre-growth annealing, droplet growth, and post-growth annealing. At the beginning of the pre-growth annealing step, the As _4_ flux to the sample surface is reduced by closing the shutter and valve of the As cell as well as the main shutter in front of the sample. For samples with pre-growth overheating, the sample temperature is set to *T*=670 °C for 120 s. Finally, the sample temperature is set to the LDE process temperature and the sample is 60 s annealed for surface smoothing. In the droplet growth step, 1.0…1.8 monolayers (ML) of Al are deposited at a flux *F*_Al_=0.4 ML/s yielding Al droplet growth [18] in Volmer-Weber mode [19]. The As flux remains either minimized with closed shutters and valve or is regulated by the valve of the As cell where both As cell and main shutters are open. During the final post-growth annealing step of *t*=180 s, the deposited droplets can be transformed into nanoholes. Droplet growth and post-growth annealing take place under constant As flux and substrate temperature.

The As flux to the sample surface is determined using the flux gauge of the Riber 32 MBE chamber at sample position. A typical flux gauge reading during MBE growth is *P*_As_=1×10^−5^ Torr. The reduction of the As flux at the beginning of pre-growth annealing yields a nearly abrupt decrease of *P*_As_ by about two orders of magnitude followed by a more slowly decrease. For a typical pre-growth annealing time of 60 s, we measure *P*_As_<7×10^−8^ Torr; for times longer than about 1200 s, we get *P*_As_<1×10^−8^ Torr. A minimum constant *P*_As_=1.3×10^−7^ Torr can be achieved using flux control via the As cell valve at open As cell and main shutters. Larger valve openings allow constant *P*_As_ up to 1.5×10^−5^ Torr.

To calibrate the flux gauge reading *P*_As_ with respect to the flux *F*_As_=*c*_1_*P*_As_ of As atoms incorporated into a growing GaAs layer, we measure As-induced reflection high-energy electron diffraction (RHEED) oscillations after Ga pre-deposition [20]. The data yield an As incorporation rate of *F*_As_=0.83 ML/s at *P*_As_=2.5×10^−6^ Torr and, thus, a value for the proportionality constant *c*_1_=3.3×10^5^ ML s ^−1^*Torr*^−1^.

## Results and Discussion

### Influence of Arsenic Flux and Surface Reconstruction

Figure 1a shows a (001) AlGaAs surface after local droplet etching at pre-growth annealing and LDE process temperature both set to *T* = 605 °C, As background pressure *P*_*As*_≃6×10^−8^ Torr, 60-s pre-growth annealing time, droplet growth during 1.4 ML Al deposition, and 180-s post-growth annealing time. Clearly visible are uniform low-density nanoholes with depth of about 40 nm. The second sample, shown in Fig. 1b, was fabricated using the same process conditions except an additional 120-s overheating step at *T*=670 °C during pre-growth annealing. Droplet deposition and post-growth annealing were performed again at *T*=605 °C. Importantly, now the resulting surface is covered mainly with droplets and only very few nanoholes are visible.

The necessity of a small As flux for the transformation of the deposited droplets into nanoholes was already observed previously [17, 18]. However, the additional influence of pre-growth overheating was not reported, so far. In order to study the overheating effect, we have recorded the RHEED pattern during this step (details are described in the next section). The RHEED data suggests that a surface reconstruction can act as a kind of reservoir for As on the surface. Together with the small As flux, this yields sufficient As for droplet etching (Fig. 1a). Overheating empties the reservoir and now the As background flux alone does not provide enough As for hole etching (Fig. 1b).

To support these findings, two additional samples are fabricated at fully minimized As pressure. After growth of the AlGaAs substrate, the samples are quenched under high As flux to maintain an As-rich surface reconstruction and stored for at least 10 h. The As pressure is reduced during this period down to *P*_*As*_≃1×10^−10^ Torr. After that, the first sample is heated to *T*=550 °C, 1.0 ML Al is deposited, followed by post-growth annealing for 180 s. The resulting surface shows uniform holes with depth of about 16 nm (Fig. 1c). In contrast, the second sample grown under equal conditions except an additional pre-growth overheating step shows droplets on the surface (Fig. 1d). These results clearly demonstrate the relevance of an As-rich surface reconstruction representing a surface As reservoir which can provide enough As to enable droplet etching even at *F*_As_≃ 0. On the other hand, overheating empties the As reservoir and the deposited droplets are conserved without As flux.

To summarize this part, the amount of As required for droplet etching can be supplied by both, a small As flux to the surface as well as by the surface reconstruction acting as As reservoir. Importantly, droplet etching processes without overheating can include both contributions.

### Evaporation of Top-Layer Arsenic

**Fig. 2:**
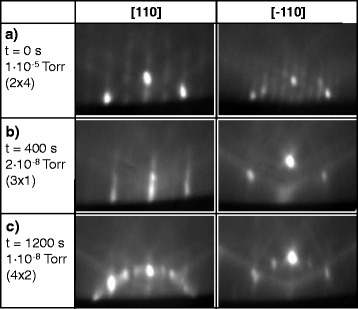
AlGaAs surface reconstructions measured with RHEED along [110] and [ −110] azimuths at *T*=600 °C after minimization of the As flux at *t*=0 s. **a** (2 ×4) reconstruction at *t*=0 s, *P*_As_=1×10^−5^ Torr. **b** (3 ×1) reconstruction at 0 s <*t*<*t*_*c*_, *P*_As_≃2×10^−8^ Torr. **c** (4 ×2) reconstruction at *t*>*t*_*c*_, *P*_As_≃1×10^−8^ Torr

In the following, the evaporation of the topmost As reservoir on a (001) AlGaAs surface is studied with reflection high-energy electron diffraction during annealing at varied temperature *T* and low As pressure. The initial surfaces for the annealing experiments are stabilized by an As flux *P*_As_=1.4×10^−5^ Torr and, thus, As-terminated. In the studied temperature range of *T*=590−650 °C in situ RHEED indicates a (2 ×4) reconstruction of the initial surface (Fig. 2a) which is expected for such conditions [21]. Assuming a *γ*(2 ×4) reconstruction [22], the initial As coverage of the topmost layer is about 1 ML.

Pre-growth annealing starts at *t*=0 s with a minimization of the As flux by closing the As cell valve and shutter as well as by closing the main shutter in front of the sample. The reconstruction changes within a few seconds from (2 ×4) to (3 ×1) (Fig. 2b), indicating now a surface As coverage smaller than 1 ML. After a critical time *t*_*c*_ with respect to the instant of As flux minimization, the reconstruction changes to (4 ×2) (Fig. 2c). This reconstruction is related to a Ga/Al-terminated surface with topmost As coverage of about zero. After re-opening the shutters and As valve, the As flux increases to its initial value *P*_As_=1.4×10^−5^ Torr and RHEED indicates a re-established As-terminated (2 ×4) reconstruction.

**Fig. 3:**
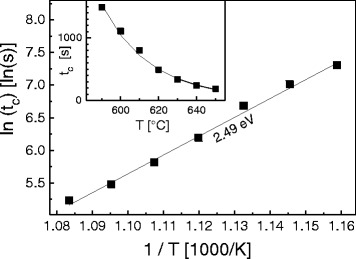
Critical time *t*_*c*_ between minimizing *F*_As_ and occurrence of a Ga/Al rich (4 ×2) reconstruction as function of sample temperature *T*. Symbols denote values measured with RHEED and lines an Arrhenius-type fit. A desorption activation energy *E*_As,*D*_=2.49 eV is determined from an Arrhenius analysis of the data

**Fig. 4:**
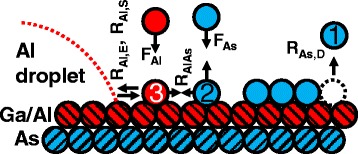
Schematic cross-section of a (001) AlGaAs surface with Ga/Al (*red*) and As (*blue*) atoms. Dashed spheres mark lattice atoms. As atom (1) illustrates desorption from the As surface reservoir. The deposited As atom (2) can re-evaporate or react with an Al adatom. The mobile Al atom (3) is generated by deposition or detachment from a droplet and can attach to a droplet or react with an As atom. The corresponding rates are discussed in the text

Figure 3 shows measured values of *t*_*c*_ as function of the substrate temperature *T*. The data establish a clear decrease of *t*_*c*_ with increasing *T*. For a quantitative analysis, we assume a thermally activated arsenic desorption rate *R*_As,*D*_=*ν* exp[−*E*_As,*D*_/(*k*_*B*_*T*)] (Fig. 4), with a vibrational frequency *ν*, an activation energy *E*_As,*D*_, and Boltzmanns constant *k*_*B*_. The experimental data are well reproduced by a thermally activated rate, where an arsenic desorption-related activation energy *E*_As,*D*_=2.49 eV is determined by an Arrhenius fit.

The As evaporation experiments establish that the amount of As stored in a surface reservoir depends on *T* and *t*. For LDE processes without overheating, a 60-s pre-growth annealing step at *T*< 660 °C will not empty the As reservoir. That means that the temperature during droplet etching influences not only the dynamics of kinetic processes but also the amount of As on the surface. On the other hand, overheating at 670 °C completely empties the As reservoir within a few seconds.

### Regimes of Arsenic Flux

**Fig. 5:**
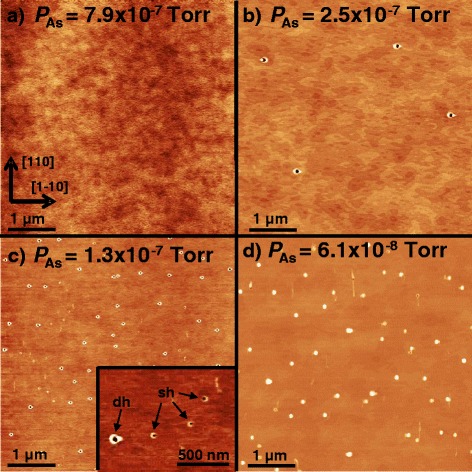
AFM images of AlGaAs surfaces after LDE with pre-growth overheating, etching at *T*=605 °C, and varied *P*_As_. **a**) *P*_As_=7.9×10^−7^ Torr. **b**) *P*_As_=2.5×10^−7^ Torr. **c**) *P*_As_=1.3×10^−7^ Torr. **d**) *P*_As_=6.1×10^−8^ Torr. The inset in **c** shows a magnification of the bimodal depth distribution with shallow (sh) and deep (dh) holes

In the following, we study droplet etching processes with pre-growth overheating (120 s at *T*=670 °C) for a complete emptying of the As reservoir and, thus, an As supply controlled only by the As flux. Figure 5 shows examples of AlGaAs surfaces after LDE with *T*=605 °C, *F*_Al_=0.4 ML/s, *θ*_Al_=1.4 ML, and varied *P*_As_ controlled by the valve of the As cell. The experiments indicate four As pressure-dependent regimes:

1. $P_{\text {As}} \gtrapprox 7.9\times 10^{-7}$ Torr, $F_{\text {As}} \gtrapprox $ 0.26 ML/s: planar growth with flat surface morphology and without nanoholes or droplets (Fig. 5a).
2. *P*_As_≃2.5×10^−7^…4.5×10^−7^ Torr, *F*_As_≃ 0.082 …0.15 ML/s: formation of uniform deep nanoholes. An example with about 16-nm-deep holes is shown in Fig. 5b.
3. *P*_As_≃1.3×10^−7^ Torr, *F*_As_≃ 0.043 ML/s: formation of holes with bimodal depth distribution [15], i.e., a high density of shallow holes has been formed in addition to the desired low-density deep holes (Fig. 5c).
4. *P*_As_≾6.1×10^−8^ Torr, *F*_As_≾ 0.02 ML/s: conservation of the initial droplets. An example is shown in Fig. 5d.

For stoichiometric growth of a planar layer in regime 1, we would expect a flux ratio *F*_Al_/*F*_As_≤1. However, the experiments indicate for the transition between planar growth and hole etching in regime 2 a higher flux ratio of *F*_Al_/*F*_As_=2±0.5. This effect might be related to the short Al deposition time, where the As flux during post-growth annealing is sufficient to incorporate the excessive Al into a planar layer.

At even more reduced *P*_As_ and, thus, increased *F*_Al_/*F*_As_ in regime 2, the amount of excessive Al is high enough for nucleation of liquid Al droplets. According to the mechanism described in [18], the droplets transform into nanoholes during post-growth annealing.

A bimodal hole depth distribution with additional shallow holes was already discussed in [15]. There, the transition between uniform and bimodal holes was observed when the Al droplet material coverage exceeds a threshold value. The present data demonstrate that this transition is observed also for a reduction of *F*_As_. This might indicate that the uniform-bimodal transition depends on the Al to As ratio. Experiments at a higher temperature indicate that the transition also depends on the temperature. Etching at *T*=630 °C and *P*_As_=4.5×10^−7^ Torr yields uniform holes whereas for *P*_As_=2.5×10^−7^ Torr a bimodal distribution is found. This means that at higher *T*, the uniform-bimodal transition shifts towards a higher As flux. A possible explanation for the additional temperature dependence might be a reduction of the As coverage due to *T*-dependent re-evaporation.

The central mechanisms distinguishing between either droplet etching or conservation (regime 4) will be discussed in the next section.

### Model of Droplet Material Removal

For nanohole formation, it is essential that the droplet material is removed during post-growth annealing [18]. The central processes for droplet shrinkage are detachment of Al atoms and subsequent reaction of these atoms with As to form AlAs on the crystal surface aside the droplets. In the following, a simple rate-equation-based model of the droplet volume evolution is proposed which allows to distinguish process parameters leading to either droplet conservation or droplet material removal (etching).

The processes considered by the model are sketched in Fig. 4. The average number *V*_*D*_ of atoms inside of a single droplet is balanced by attachment of mobile Al atoms with rate $n_{\text {Al}} R_{\text {Al},S} V_{D}^{1/3}$ and detachment from the droplet boundary with rate $R_{\text {Al},E} V_{D}^{1/3}$, where *n*_Al_ is the density of mobile Al atoms on the surface, *R*_Al,*S*_=*ν* exp[−*E*_Al,*S*_/(*k*_*B*_*T*)] is the Al surface diffusion coefficient, *R*_Al,*E*_=*ν* exp[−*E*_Al,*E*_/(*k*_*B*_*T*)] is the rate at which single Al atoms escape from a droplet, and *E*_*Al*,*S*_ and *E*_*Al*,*E*_ are activation energies. *ν*=2*k*_*B*_*T*/*h* is a vibrational frequency [23], with Boltzmann’s constant *k*_*B*_ and Planck’s constant *h*. This yields for the volume evolution of a single droplet in units of the number of atoms inside the droplet

1 $$ \frac{d V_{D}}{dt} = n_{\text{Al}} R_{\text{Al},S} V_{D}^{1/3} - R_{\text{Al},E} V_{D}^{1/3}  $$

The present approach does not consider droplet nucleation and according to previous experimental results [15] we assume a deposition-time independent droplet density *N*_*D*_.

Al and As atoms impinge with fluxes *F*_Al_ and *F*_As_ on the surface which increases the respective adatom densities. Additional processes related to the dissociation of impinging As _4_ molecules [24] are not considered. Mobile Al atoms can react with mobile As atoms to form AlAs. The Al-As reaction rate is *R*_AlAs_=*ν* exp[−*E*_AlAs_/(*k*_*B*_*T*)], with the activation energy *E*_AlAs_. Al adatoms can also attach to droplets with rate characterized by the Al surface diffusion coefficient *R*_Al,*S*_, or detach from droplets with the escape rate *R*_Al,*E*_. The corresponding time evolution of the Al adatom density is

2 $$ \begin{aligned} \frac{\mathrm{dn_{Al}}}{\text{dt}} = \ & F_{\text{Al}} - n_{\text{Al}} n_{\text{As}} R_{\text{AlAs}} - n_{\text{Al}} R_{\text{Al},S} N_{D} V_{D}^{1/3} + \\ & R_{\text{Al},E} N_{D} V_{D}^{1/3} \end{aligned}  $$

The evolution of the deposited As adatom density is

3 $$ \frac{\mathrm{d n_{As}}}{\text{dt}} = F_{\text{As}} - n_{\text{Al}} n_{\text{As}} R_{\text{AlAs}}  $$

For simplicity, re-evaporation of arsenic is neglected here since the critical times in Fig. 3 are much longer than the post-growth annealing time.

**Fig. 6:**
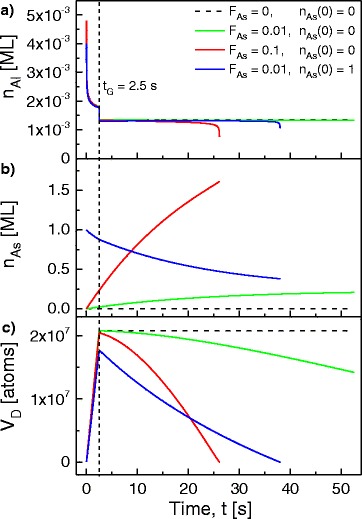
Calculated time-dependent **a** Al adatom density *n*_Al_, **b** As adatom density *n*_As_, and **c** droplet volume *V*_*D*_. The process parameters are *T*=605 °C, *N*_*D*_=4.8×10^8^, and varied *F*_As_ and *n*_As_(0) as indicated. *F*_Al_=0.4 ML/s for *t*<*t*_*G*_=2.5 s (growth) and *F*_Al_=0 for *t*>*t*_*G*_ (annealing). The As flux *F*_As_ in ML/s and the initial As adatom density *n*_As_(0) in ML are varied as indicated

Model calculations for droplet growth and post-growth annealing are performed by numerically solving Eqs. 1–3 with the initial conditions *V*_*D*_(0)=0,*n*_Al_(0)=0, and *n*_As_(0)= 0 or 1 ML. As an example, Fig. 6 shows calculation results for *T*=605 °C, *F*_Al_= 0.4 ML/s during growth and zero during annealing, *t*_*G*_=2.5 s, and *N*_*D*_=3×10^7^ cm ^−2^. *F*_As_ and *n*_As_(0) are varied as indicated. The chosen model parameters are *E*_Al,*S*_=1.0 eV, *E*_Al,*E*_= 1.5 eV, and *E*_AlAs_=2.1 eV.

The Al adatom density *n*_Al_ increases abruptly after growth starts at *t*=0 (Fig. 6a). Subsequently, *n*_Al_ decreases continuously due to attachment of Al adatoms to the droplets. After growth stops at *t*=*t*_*G*_, *n*_Al_ drops to a new equilibrium value and remains constant until the droplets become very small. Interestingly, there is no significant influence of *F*_As_. The As adatom density *n*_As_ shows a continuous increase (*n*_As_(0)= 0) or decrease (*n*_As_(0)= 1 ML) with time and a very strong influence of *F*_As_ (Fig. 6b). The droplet volume increases linearly during growth (Fig. 6c). During annealing, *V*_*D*_ decreases with slope strongly dependent on *F*_As_ and *n*_As_(0).

In view of the experimental results, the constant or slowly decreasing *V*_*D*_ calculated for *n*_As_(0)=0,*F*_As_=0 and 0.01 ML/s (dashed and green lines in Fig. 6c) corresponds to As pressure regime 4, where the initial droplets are conserved (Fig. 5d). At higher *F*_As_=0.1 ML/s, the droplet material is removed within 25 s by detachment and reaction with As (red line in Fig. 6c). Here, formation of nanoholes is expected in agreement with regime 2 (Fig. 5b). These examples illustrate how *F*_As_ can switch between droplet etching and conservation.

In addition, also the influence of an As reservoir for processes without pre-growth overheating is modeled by an initial As adatom density *n*_As_(0)=1 ML. The blue line in Fig. 6c indicates droplet material removal and, thus, hole etching at *F*_As_=0.01, *n*_As_(0)=1 ML, in agreement with Fig. 1a. This is compared to the green line with the same *F*_As_ but *n*_As_(0)=0 and droplet conservation, in agreement with Fig. 1b.

### Droplet and Hole Densities

A comparison of Fig. 1a, b as well as of Fig. 1c, d indicates an always significantly higher density of the droplets than for the nanoholes. This opens the question, which density represents the initial density of the deposited droplets. Using time-dependent experiments during Ga LDE, we have shown that coarsening by Ostwald ripening [25] reduces the droplet density before drilling [12]. We assume a similar situation for the present experiments with Al droplets. Accordingly, the higher densities in the droplet conservation regime (Fig. 1b, d) are expected to represent the densities of the initially deposited droplets. Assuming a broad initial droplet size distribution, small droplets will disappear and only large droplets will transform into deep nanoholes. In this picture, the bimodal hole depth distribution in Fig. 5c might represent an intermediate stage, where the small initial droplets are not fully coarsened, but instead transform into shallow holes.

## Conclusions

The arsenic background, essential for local droplet etching of nanoholes in AlGaAs surfaces, can be supplied by a small As flux and an As-rich surface reconstruction acting as a reservoir. Most previous experiments on LDE have been performed under process conditions, where both contributions are included. Pre-growth overheating allows to empty the As reservoir and to study the influence of the As flux during LDE under well-controlled conditions. A model of the droplet volume evolution is proposed that quantitatively reproduces the experimental data of the arsenic controlled switching between droplet etching or conservation.

## Abbreviations

LDE: Local droplet etching
MBE: Molecular beam epitaxy
ML: Monolayers
RHEED: Reflection high-energy electron diffraction
